# On‐Surface Formation of Cyano‐Vinylene Linked Chains by Knoevenagel Condensation

**DOI:** 10.1002/chem.202103094

**Published:** 2021-10-21

**Authors:** Kwan Ho Au‐Yeung, Tim Kühne, Daniel Becker, Marcus Richter, Dmitry A. Ryndyk, Gianaurelio Cuniberti, Thomas Heine, Xinliang Feng, Francesca Moresco

**Affiliations:** ^1^ Center for Advancing Electronics Dresden (cfaed) TU Dresden 01062 Dresden Germany; ^2^ Institute of Molecular Functional Materials Faculty of Chemistry and Food Chemistry TU Dresden 01062 Dresden Germany; ^3^ Institute for Materials Science TU Dresden 01062 Dresden Germany; ^4^ Theoretical Chemistry TU Dresden 01062 Dresden Germany; ^5^ Institute of Resource Ecology Helmholtz-Zentrum Dresden-Rossendorf Leipzig Research Branch 04316 Leipzig Germany; ^6^ Department of Chemistry Yonsei University Seoul Republic of Korea

**Keywords:** density functional calculations, Knoevenagel condensation, on-surface chemistry, scanning probe microscopy

## Abstract

The rapid development of on‐surface synthesis provides a unique approach toward the formation of carbon‐based nanostructures with designed properties. Herein, we present the on‐surface formation of CN‐substituted phenylene vinylene chains on the Au(111) surface, thermally induced by annealing the substrate stepwise at temperatures between 220 °C and 240 °C. The reaction is investigated by scanning tunneling microscopy and density functional theory. Supported by the calculated reaction pathway, we assign the observed chain formation to a Knoevenagel condensation between an aldehyde and a methylene nitrile substituent.

## Graphical abstract

**We present** the thermally induced synthesis of CN‐substituted phenylene vinylene chains through Knoevenagel condensation reaction on an Au(111) surface. The formation of C=C interlinked chains is investigated by low‐temperature scanning tunnelling microscopy and density functional theory.

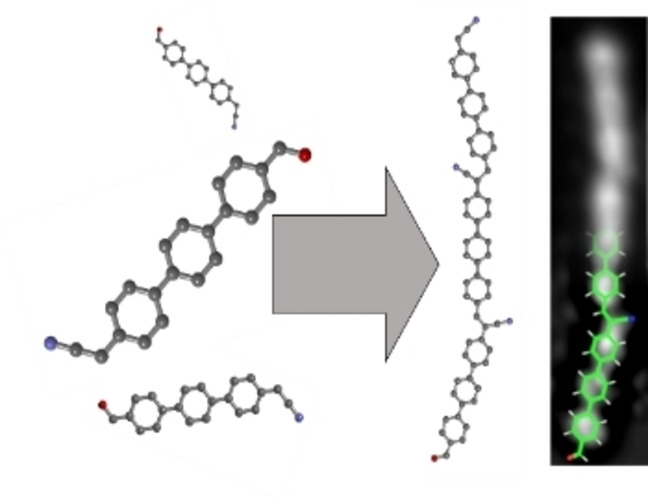

On‐surface synthesis is a fast developing field of research^[1]^ for exploring a wide spectrum of nanostructures that cannot be achieved by conventional solution chemistry,[^2^, ^3^] for examples, graphene nanoribbons (GNRs),[^4^, ^5^] long acenes,[^6^, ^7^] 2D conjugated polymers or porous graphene structures,^[8]^ and functional molecular machines.[^9^, ^10^] Among several successfully tested on‐surface reactions, Ullmann coupling with covalent C−C bond formation remains the preferred pathway for inducing on‐surface coupling of molecular precursors.^[8]^ This reaction between aryl halides, however, limits the choice of moieties and structures, and hence the corresponding functionalities. Nonetheless, only a few studies involve the formation of carbon‐carbon double bonds (C=C)[^11^, ^12^, ^13^, ^14^, ^15^, ^16^] or triple bonds (−C≡C−).[^17^, ^18^, ^19^] Therefore, the synthetic toolbox requires substantial expansion in order to make this technique a competitive method of surface chemistry.

The incorporation of C=C bonds into conjugated nanostructures with additional functional groups (for example, nitrogen substitution) has attracted extensive attention in recent years due to their intriguing electronic properties.[^20^, ^21^, ^22^, ^23^, ^24^] In aqueous solution, an efficient pathway for synthesizing CN‐substituted vinylene‐linked polymers coupling with C=C bond is Knoevenagel polycondensation between aldehydes and methylene nitriles with an excess of strong bases, such as tetrabutylammonium hydroxide and potassium *tert*‐butoxid.[^25^, ^26^] In particular, the introduction of electron‐withdrawing CN‐groups in poly(*p*‐phenylenevinylene) (PPV) leads to a twisted geometry, a reduced energy level of the lowest unoccupied molecular orbital (LUMO), and an increased electron‐affinity.[^27^, ^28^, ^29^] Recent studies on fabricating luminescent organic materials with, for example, high photo stability through the Knoevenagel polymerization have been reported.[^30^, ^31^] The Knoevenagel reaction between two different precursors was performed at the liquid‐solid and vapor‐solid interfaces.[^32^, ^33^]

In the present work, we report the formation of CN‐substituted phenylene vinylene chains on the Au(111) surface. We propose that the chain formation is driven by a thermally‐induced Knoevenagel polycondensation (Scheme 1). The precursor 4‐formyl(4,4’’‐terphenyl)‐4’’‐methylenenitrile **(1)** and the target conjugated oligo‐phenylenevinylenes **(3)** are investigated by low‐temperature scanning tunneling microscopy (STM) under ultra‐high vacuum (UHV) conditions. Density functional theory (DFT) calculations confirmed adsorption geometries and the structure of the conjugated chains. The proposed reaction pathway is theoretically supported by the nudged elastic band (NEB) method.

**Scheme 1:**
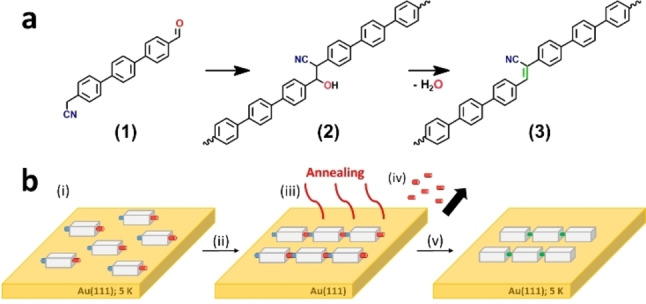
Schematic formation of CN‐substituted phenylene vinylene chains on surface under UHV conditions. a) Reaction pathway for the Knoevenagel condensation. b) Proposed schematic illustration of the Knoevenagel condensation reaction on the Au(111) surface. i) Thermal deposition of the precursors on Au(111). ii) Diffusion of the precursors. iii) Annealing of the surface to activate coupling and condensation. Intermediate linkages are formed. iv) Chains undergo dehydration, and the water molecules spontaneously desorb from the surface upon annealing. v) Final products (See Supporting Information for the synthesis steps).

Precursor molecules **(1)** (Scheme 1a), were deposited thermally (*T*_evap_=170 °C) on the clean Au(111) surface kept at room temperature under UHV conditions at a coverage of about 0.9 ML as determined by STM images. STM experiments were performed after cooling the sample to *T*=5 K.

After deposition, self‐assembled and densely packed monolayer islands of the precursors can be observed in the STM images (Figures 1a and S10). The molecules can be clearly identified as the linear features oriented in parallel. Different spacing between the molecules is observed possibly due to both the mismatch to the Au(111) lattice and the different intermolecular interactions (that is, van der Waals interactions) at the methylene nitrile and aldehyde ends.

**Figure 1:**
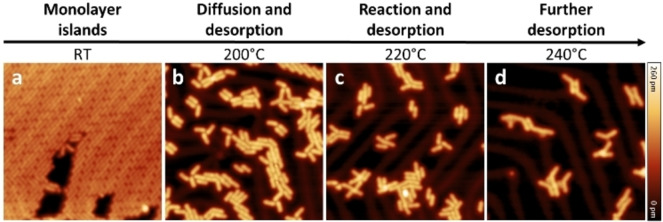
Overview STM images (size: 23 nm×23 nm) from stepwise annealing preparations. a) Formation of monolayer islands after thermal deposition of the precursor (**1**) on the Au(111) surface kept at RT. b–d) Stepwise growth of the phenylene vinylene chains after annealing at b) 200 °C, c) 220 °C, and d) 240 °C. STM images are taken in constant current mode under a) *V*=300 mV and *I*=3 pA, b) *V*=500 mV and *I*=5 pA; c, d) *V*=500 mV and *I*=10 pA.

To induce the formation of molecular chains, we annealed stepwise the adsorbed precursors. After each annealing step, the sample was cooled down to *T*=5 K and STM images were recorded. The first annealing step at *T*=200 °C causes partial desorption and molecular diffusion. The monolayer islands deform into parallel structures, still formed by individual precursors (Figure 1b). After annealing at 220 °C, the coverage decreases sharply, and chains of molecules are visible through STM (Figure 1c). As commonly observed in on‐surface synthesis, the ordered islands of weakly bonded single precursors progressively evolve into disordered rigid structures.[^8^, ^33^]

The last annealing step at 240 °C (Figure 1d) causes further desorption of monomers and short chains, with a slight improvement of the average chain lengths (See Figure S9 for a statistical analysis). At this stage, only the kinks of the Au(111) reconstruction sites are still populated with molecular chains (similar results are obtained by slow precursors deposition on the hot surface, see Figure S8). Furthermore, after annealing at *T*≥220 °C, further structures probably due to van der Waals or metalorganic bonds with Au adatoms are visible.^[34]^ Figure 2(a) displays a STM image obtained after annealing precursors **(1)** to 240 °C. Chains of different length are visible, while the oligomers show a twisted geometry at the CN‐vinylene linkages between the phenylene motifs due to steric hindrance.^[35]^

**Figure 2:**
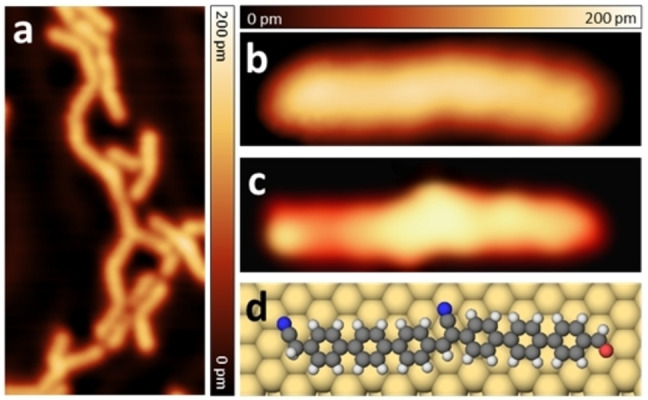
STM images after preparation of the precursors **(1)** on Au(111) at 240 °C. a) Close‐up STM image (size: 15.5 nm×7.8 nm) of an assembly of different lengths of oligomers. All STM images are taken under *V*=500 mV and *I*=5 pA. B–d) Comparison of experimental, simulated STM images and the corresponding calculated adsorption geometry of a phenylene vinylene dimer. b) Experimental STM image (size: 3.8 nm×1.1 nm) of a dimer is taken under *V*=500 mV and *I*=5 pA. c) Simulated STM image (size: 3.8 nm×1.1 nm) of a dimer under *V*=500 mV. d) Corresponding calculated adsorption geometry (top view) of Figure (c).

In Figure 2(b), the STM image of a dimer is compared with a simulated STM image (Figure 2c), extracted from the adsorption geometry calculated by DFT (Figure 2d). The experimental and simulated STM images are in good agreement confirming the formation of short covalent chains.

We propose that a thermally induced Knoevenagel condensation following the reaction pathway of Scheme 1 may have happened on surface after annealing at a temperature between 220 °C and 240 °C. Following Scheme 1(a), the aldehyde and methylene nitrile substituents of the precursor molecules **(1)** could approach each other and react, then form a covalent bond **(2)**. Spontaneous dehydration is expected to obtain the chain **(3)** as a final product. The formation of intermediate **(2)** and the elimination of water molecules cannot be imaged by STM, because the annealing step takes place outside the microscope, however suggesting that the thermal energy upon annealing triggers the water desorption and the final reaction step.

We have recorded high‐resolution STM images of the formed chains. Figure 3(a) presents a STM image of a trimer with three interlinked phenylene vinylene units measured with a CO‐functionalized tip in constant height mode. The bright round lobes clearly show the 9 individual phenyl rings. We calculated by DFT the adsorption structure of a chain formed by three monomers. The calculated relaxed structure is superposed to the STM image in Figure 3(b). Figure 3(c) reports the calculated adsorption geometry on Au(111). The good agreement between experiment and theory confirms the formation of the final product **(3)**. From the detailed calculations of the chemical reaction (presented below for a dimer), it follows that the distance between the phenylene units is much shorter for covalently bonded structures than for unbonded ones. Therefore, comparing the calculated structure with the experimental image, one can distinguish between bonded and unbonded structures. Please also notice that STM does not resolve the CN groups at the end or in the chain. Furthermore, the chains are flexible on the surface, showing different possible adsorption geometries and orientations (see also Figures 1d, 2a, 3 and S8).

**Figure 3:**
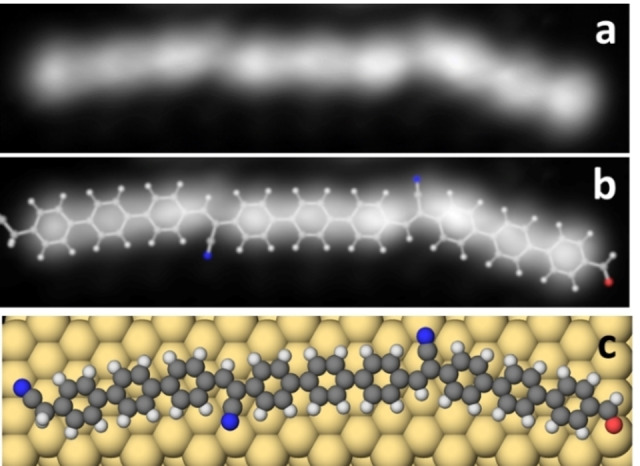
Structure of a CN‐vinylene‐linked trimer. a) Constant height high‐resolution STM image (size: 4.6 nm×1.1 nm, *V*=10 mV). b) The corresponding calculated trimer structure overlaid on (a). c) The corresponding calculated adsorption geometry of (a) on the Au(111) surface.

To understand the mechanism of the chain formation, we calculated the energy reaction path for the dimer formation by DFT and NEB method (Figure 4). We found the initial (**I**) and final (**VI**) states from the Born‐Oppenheimer potential energy and performed the NEB calculations to find the reaction path. To model the experimental conditions, the initial state is represented by two precursor molecules **(1)** adsorbed on the Au(111) surface with the methylene nitrile and aldehyde groups facing each other respectively. The final state (**VI**) with phenylene vinylene moieties and dissociated water molecule has an energy about 0.55 eV lower than the initial state (**I**). The reaction undergoes the intermediate states (**II**–**V**), where the new C−C bond is formed, and one water molecule is dissociated. The dashed lines show the intermediate states that were calculated but not shown. In particular, the state between **II** and **III** is an important intermediate local energy minimum lowering the maximum energy barrier from about 2 eV (between **I** and **III**) to 1.2 eV (between **I** and **II**). Such value is comparable with literature values for on‐surface reactions at similar conditions.^[36]^ Furthermore, we note that the excitation energy necessary to trigger the condensation (0.25 eV from **IV** to **V** in Figure 4) is much lower than the energy needed from step **I** to **II** (1.2 eV). It is therefore reasonable to assume that the thermal energy upon annealing immediately triggers the final reaction step and, hence, it is not possible to observe intermediate structures (**IV** in Figure 4) in the STM images after annealing.

**Figure 4:**
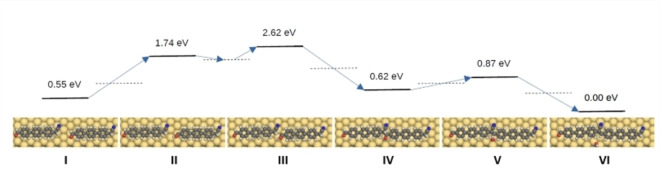
Energy profiles at different states and the corresponding adsorption geometries. Top view of the simulated adsorption geometries, calculated by DFT, starting from the precursors (state I), through intermediate states (II–V) to the final molecule (state VI). The corresponding reaction energy profiles (in eV) are calculated employing the nudged elastic band (NEB) method.

For geometry optimization and NEB calculations, we used the DFT method as implemented in the CP2 K software package (cp2k.org) with the Quickstep module.^[37]^ We applied the Perdew‐Burke‐Ernzerhof exchange‐correlation functional,^[38]^ the Goedecker‐Teter‐Hutter pseudo‐potentials^[39]^ and the valence double‐*ζ* basis sets, in combination with the DFT−D3 method of Grimme^[40]^ for van der Waals correction. The calculations of STM topography images were performed by the DFTB+XT code from TraNaS OpenSuite (tranas.org/opensuite), partially based on the DFTB+[^41^, ^42^] software package. We also used the density functional based tight‐binding method with auorg‐1‐1 parametrization[^43^, ^44^] as implemented in the DFTB+ package. We considered a realistic atomistic system including the STM tip and the substrate, both connected to semi‐infinite electrodes. The simulation of STM images in the constant‐current mode was done based on the current calculations by the Green function technique.^[45]^ The data is analyzed, and the images are made by the PyMOL Molecular Graphics System, Version 2.4 open‐source build, Schrödinger, LLC.

In summary, we have presented the on‐surface synthesis of CN‐substituted phenylene vinylene chains on the Au(111) surface through the Knoevenagel condensation. A combination of STM images and calculations confirms and provides insights on the proposed mechanism, allowing a quantitative understanding of the coupling steps energetically. Our work introduces a new possible reaction route into the toolbox of on‐surface synthesis, and represents a further step towards a property‐oriented on‐surface synthesis based on the rational design of monomers and polymerization paths. Further work is now needed to optimize the reaction parameters and improve the precursor design as well as yield in a quantitative way.

## Conflict of interest

The authors declare no conflict of interest.

## Supporting information

As a service to our authors and readers, this journal provides supporting information supplied by the authors. Such materials are peer reviewed and may be re‐organized for online delivery, but are not copy‐edited or typeset. Technical support issues arising from supporting information (other than missing files) should be addressed to the authors.

Supporting Information
