# Supplementary material for: On‐Surface Formation of Cyano‐Vinylene Linked Chains by Knoevenagel Condensation
Source: Chemistry. 2021 Oct 21;27(69):17336–40. doi: 10.1002/chem.202103094 (PMC9298206; doi:10.1002/chem.202103094)
Supplement: Supplementary file 1 — Supporting Information [file CHEM-27-17336-s001.pdf]

Chemistry–A European Journal

Supporting Information

On-Surface Formation of Cyano-Vinylene Linked Chains by Knoevenagel Condensation

Kwan Ho Au-Yeung, Tim Kühne, Daniel Becker, Marcus Richter, Dmitry A. Ryndyk, Gianaurelio Cuniberti, Thomas Heine, Xinliang Feng, and Francesca Moresco*

Table of Contents

Synthesis of precursor 2

Characterizations of precursor 5

STM experimental details 9

Additional STM images 9

References 12

Synthesis of precursor

General information

Unless otherwise stated, the commercially available reagents and dry solvents were used without further purification. The reactions were performed using standard vacuum-line and Schlenk techniques, work and purification of all compounds were performed under air and with reagent-grade solvents. Column chromatography was done with silica gel (pore size 60 Å, 70 - 230 mesh, 63 – 200 μm from Sigma Aldrich) and silica coated aluminum sheets with fluorescence indicator from Merck were used for thin layer chromatography. Purification by recycling gel permeation chromatography (rGPC) was performed on JAI HPLC LC 9110 II NEXT with fraction collector FC- 3310 and GPC columns 2H and 1H (connected in series). The rGPC was used with HPLC-grade chloroform at room temperature.

Nuclear magnetic resonance (NMR) spectroscopy: All ^{1}H, ^{13}C- and 2D-NMR measurements were recorded on a BRUKER AVANCE III 300 at 25°C using standard pulse programs. Chemical shifts are reported as δ-values in ppm. Coupling constants (J) are given in Hertz (Hz). Chemical shift was referenced in regards to δ chloroform-d_1 (δ (^{1}H) = 7.26 ppm, δ (^{13}C) = 77.16 ppm). The following abbreviations are used to describe peak patterns as appropriate: s = singlet, d = doublet, m = multiplet.

Mass spectrometry: High-Resolution Atmospheric Pressure Chemical Ionization (APCI) mass spectra was recorded with Agilent 6538 Ultra High Definition (UHD) Accurate-Mass QTOF LC/MC system, using the positive mode.

Overview

The precursor (**1**), namely 4-formyl(4,4’’-terphenyl)-4’’-methylenenitrile, was synthesized in two step synthesis starting from the commercially available 1-bromo-4-iodobenzene (**4**). Firstly, the selective Suzuki cross-coupling of 4'-bromo-[1,1'-biphenyl]-4-carbaldehyde (**6**) was carried out from 1-bromo-4-iodobenzene (**4**) and 4-(formyl)benzeneboronic acid (**5**) in 63% yield. Then, a second Suzuki cross-coupling between intermediate (**6**) and 4-(cyanomethyl)benzeneboronic acid (**7**) afforded the precursor (**1**) in 49 % yield. The chemical structure of precursor (**1**) was confirmed by NMR spectroscopy and high-resolution electrospray ionization mass spectrometry (HR-ESI-MS)

Table S1. List of the chemicals inclusive suppliers and purity.

Chemical Name	Suppliers	Purity
**1-Bromo-4-iodobenzene**	ABCR	97%
**4-(Formyl)benzeneboronic acid**	Manchester Organics	97%
**4-(Cyanomethyl)benzeneboronic acid**	Manchester Organics	95%

Synthesis of 4'-bromo-[1,1'-biphenyl]-4-carbaldehyde (6)

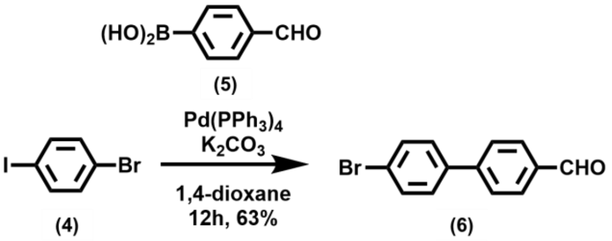

1-Bromo-4-iodobenzene (**1**, 1.00 g, 3.53 mmol, 2.00 eq.), 4-(formyl)benzeneboronic acid (**4**, 265.00 mg, 1.77 mmol, 1.00 eq.), 2*M* potassium carbonate (2.44 g, 17.67 mmol, 10.00 eq., 8.84 mL) and 75 mL 1,4-dioxane were added and purged with nitrogen gas under vigorous stirring. After 30 minutes, the tetrakis(triphenylphosphine)palladium(0) (102.12 mg, 0.09 mmol, 0.05 eq.) was added in to the reaction mixture under nitrogen counter current. The reaction mixture was heated under reflux for 12 hours. After cooling down to room temperature, the reaction mixture was quenched by the addition of water (25 mL) and extracted with dichloromethane (50 mL) three times. The collected organic layers were washed with saturated sodium chloride solution (25 mL) and dried over magnesium sulfate. The solvent was removed under reduced pressure and the residue was purified by column chromatography on silica (dichloromethane: *n*-hexane: 1/1) to afford 4'-bromo-[1,1'-biphenyl]-4-carbaldehyde (**6**). Intermediate (**6**) was obtained as colorless solid in a yield of 63 %.

NMR spectroscopy and HR-MS of (**6**) was in coincident with previous reports.[1, 2]

Synthesis of 4-formyl(4,4’’-terphenyl)-4’’-methylenenitrile (1)

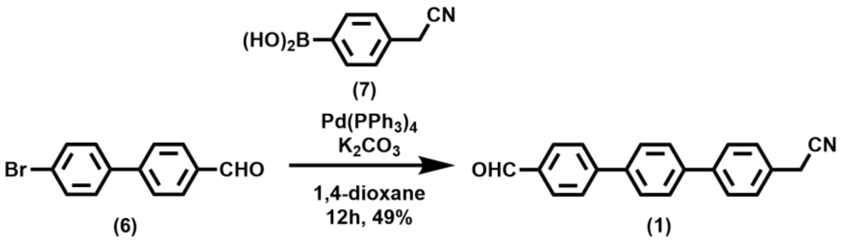

4-Bromo-[1,1'-biphenyl]-4-carbaldehyde (**6**, 0.20 g, 0.77 mmol, 1.00 eq.), 4-(cyanomethyl)benzeneboronic acid (**7**, 246.58 mg, 1.53 mmol, 2.00 eq.), 2*M* potassium carbonate (1.06 g, 7.67 mmol, 10.00 eq., 3.83 mL) and 50 mL 1,4-dioxane were added and purged with nitrogen gas under vigorous stirring. After 30 minutes, the tetrakis(triphenylphosphine)palladium (0) (44.26 mg, 0.04 mmol, 0.05 eq.) was added in to the reaction mixture under nitrogen counter current. The reaction mixture was heated under reflux for 12 hours. After cooling down to room temperature, the reaction mixture was quenched by the addition of water (15 mL) and extracted with dichloromethane (25 mL) three times. The collected organic layers were washed with saturated sodium chloride solution (10 mL) and dried over magnesium sulfate. After the removal of the solvent under reduced pressure, the crude product was filtered over a silica pad (dichloromethane: *n*-hexane: 1/1) and subsequently recrystallized from isopropanol. The residue was purified by rGPC in chloroform to obtain compound (**1**) as colorless solid (112.4 mg, 0.38 mmol) in 49%.

4-Formyl(4,4’’-terphenyl)-4’’-methylenenitrile (**1**)

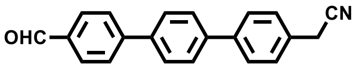

(1)

^{1}H NMR (300 MHz, $CDCl_3$): δ = 10.08 (s, 1H), 7.98 (d, *J* = 8.1 Hz, 2H), 7.81 (d, *J* = 8.1 Hz, 2H), 7.76 – 7.65 (m, 6H), 7.44 (d, J = 8.0 Hz, 1H), 3.82 (s, 2H).

^{13}C NMR (75 MHz, $CDCl_3$): δ = 192.01, 146.65, 140.47, 140.39, 139.10, 135.47, 130.50, 129.42, 128.67, 128.02, 127.92, 127.81, 127.71, 117.88, 23.53.

HR-MS (APCI-MS): m/z (M^+) = 297.1147, calcd. for $C_{21}H_{16}NO$: m/z = 297.1153, error = -2.1 ppm.

Characterizations of precursor

Mass spectroscopy

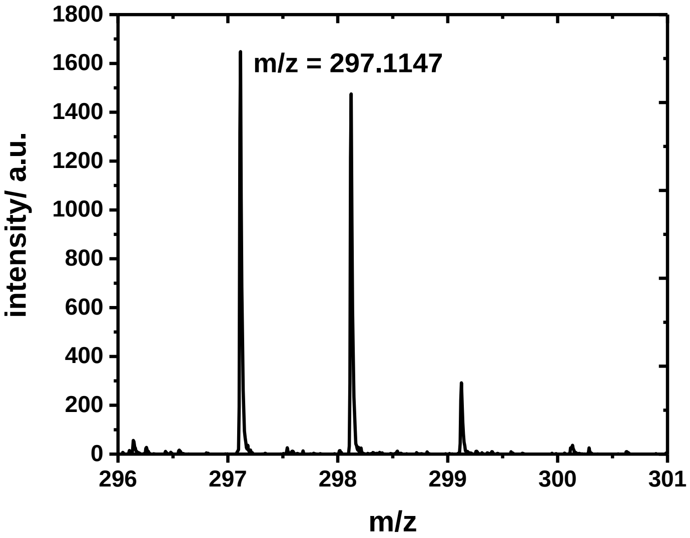

Figure S1. HR-APCI spectrum of **1**.

NMR spectroscopy

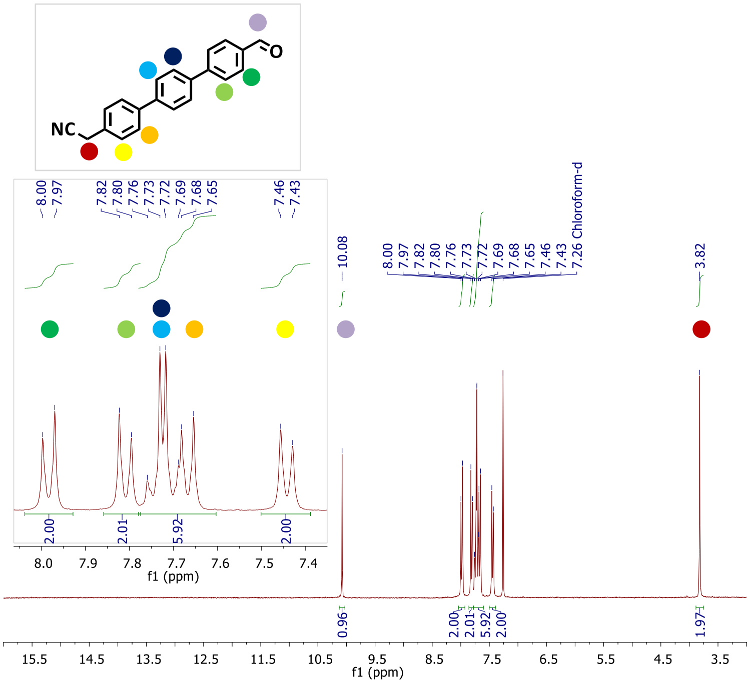

Figure S2. ^{1}H-NMR spectrum of **1** at 25°C in $CDCl_3$.

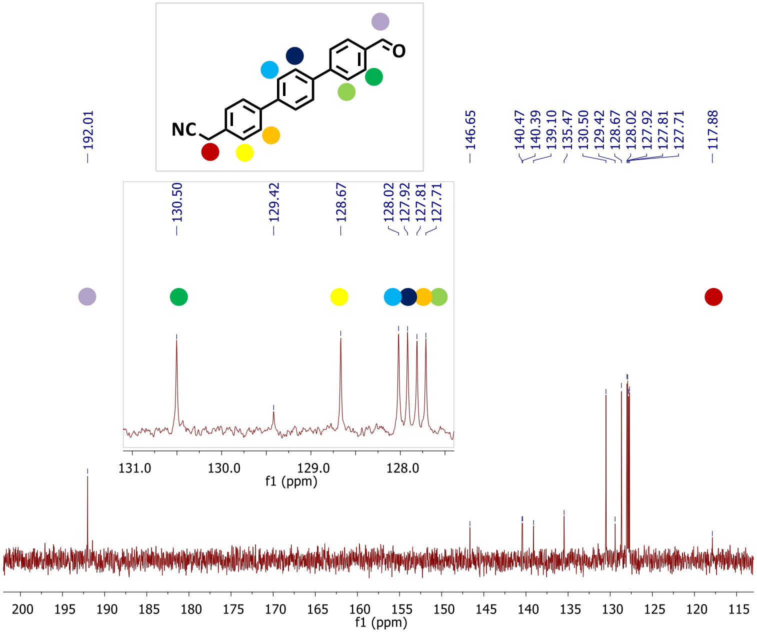

Figure S3. ^{13}C-NMR spectrum of **1** at 25°C in $CDCl_3$.

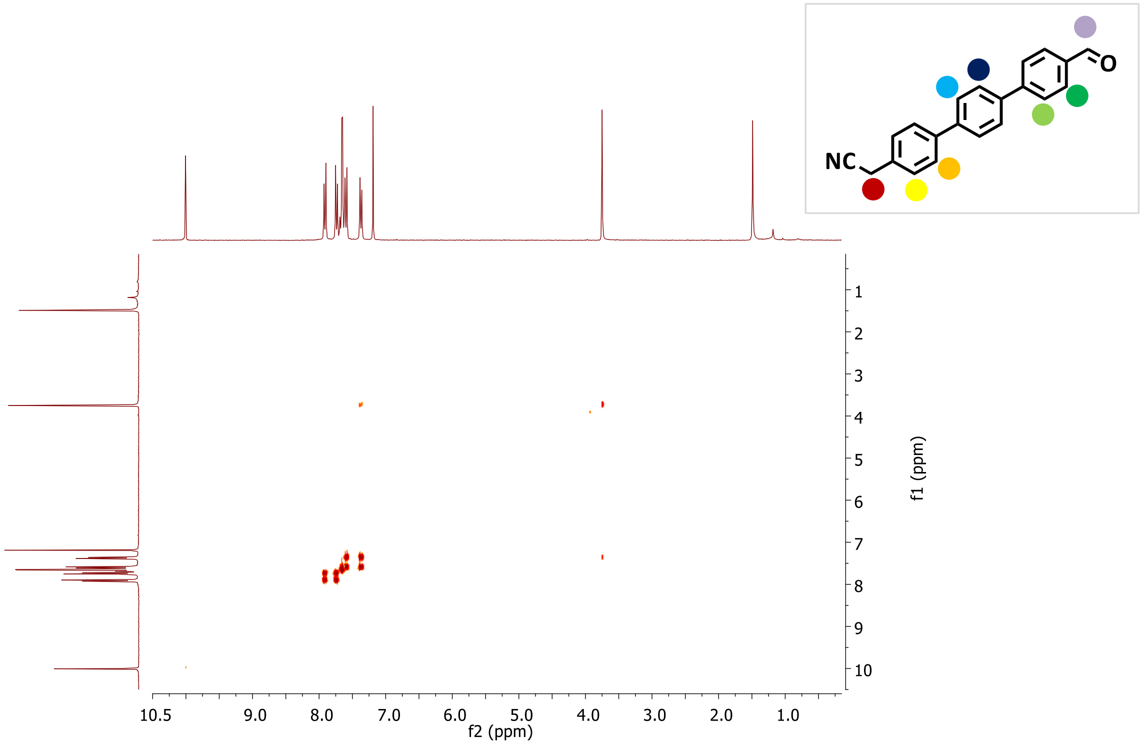

Figure S4. COSY-2D-NMR spectrum of **1** at 25°C in $CDCl_3$.

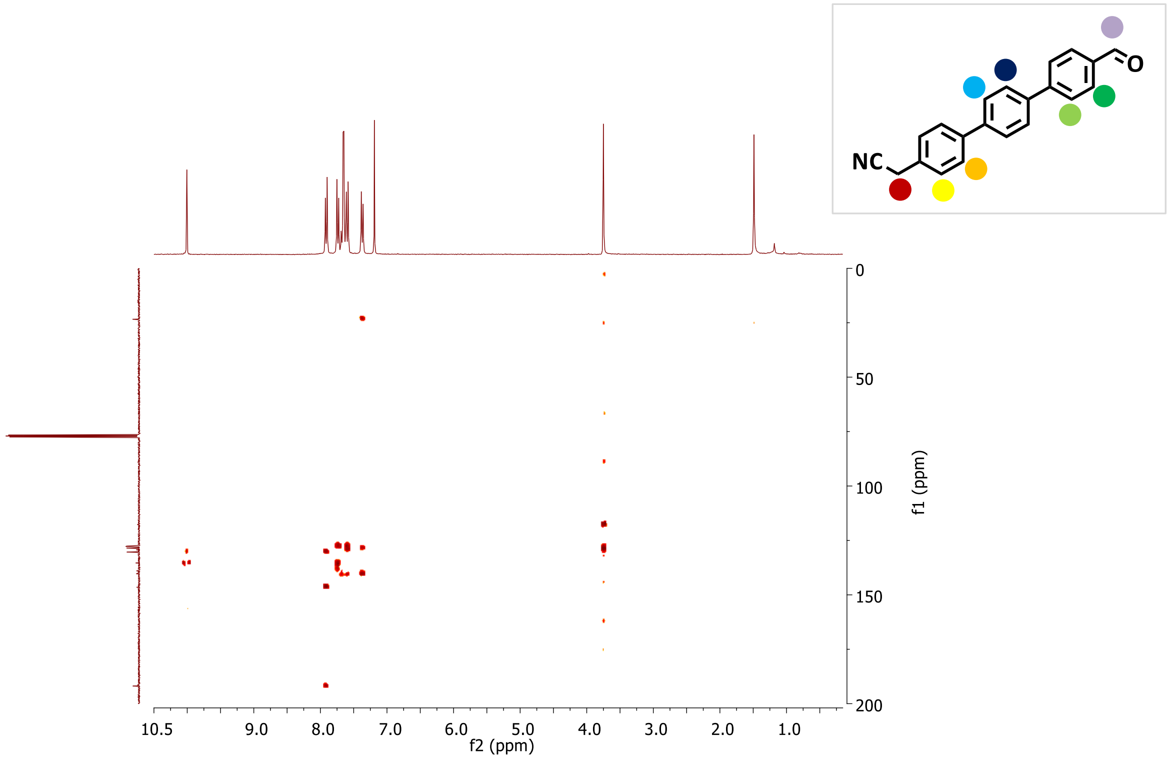

Figure S5. HMBC-2D-NMR spectrum of **1** at 25°C in $CDCl_3$.

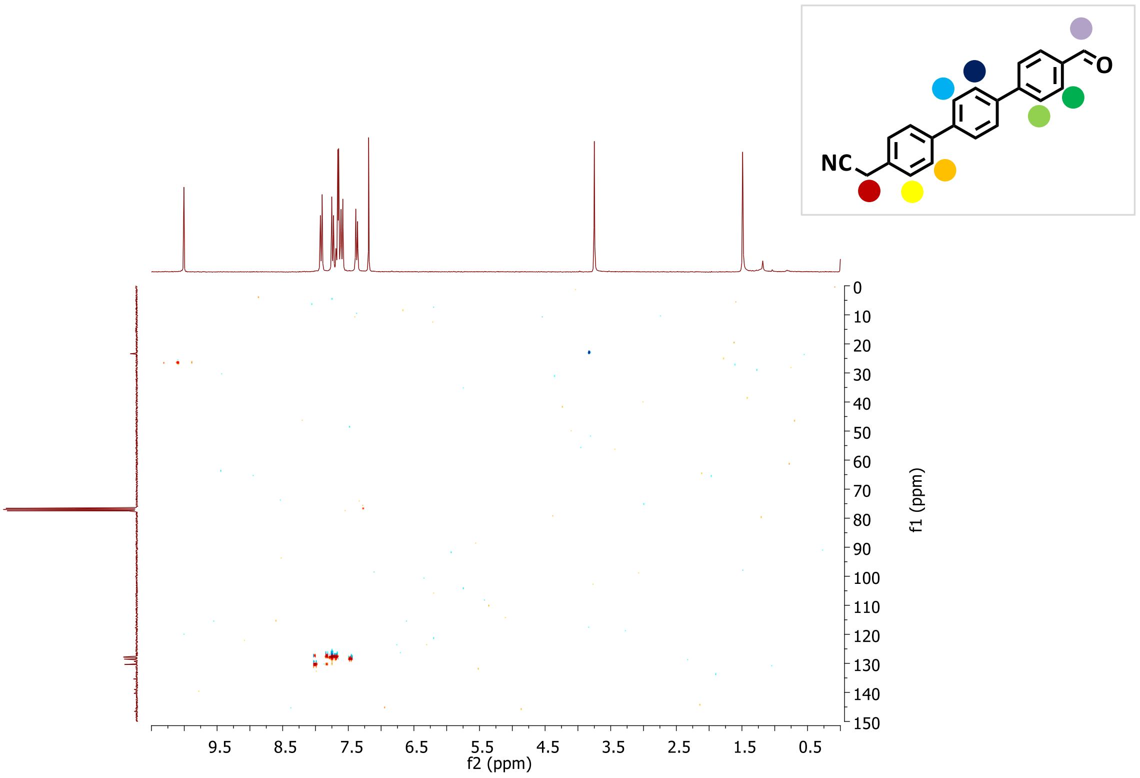

Figure S6. HSQC-2D-NMR spectrum of **1** at 25°C in $CDCl_3$.

STM experimental details

Before thermal deposition of molecules, the samples were cleaned by subsequent cycles of Ar^+ sputtering and annealing to 450°C. Precursors were deposited thermally on the clean Au(111) surface. For activating the proposed reaction, the surface was annealed stepwise, ranging from 200°C to 250°C. After each step of annealing, the sample was cooled down to 5 K (-268°C) for STM imaging. Each annealing period was 5 min at the final temperature, after a ramping phase with an average speed of 1.5°C/s.

STM experiments were performed using a custom-built instrument operating at low temperature of T = 5 K under ultrahigh vacuum ($p \approx 1 \times 10^{-10}$ mbar). STM images were recorded in constant current mode with the bias voltage applied to the sample if not specifically mentioned. High-resolution STM images were acquired with a CO-functionalized tip in constant height mode.

Additional STM images

Further CO-tip image

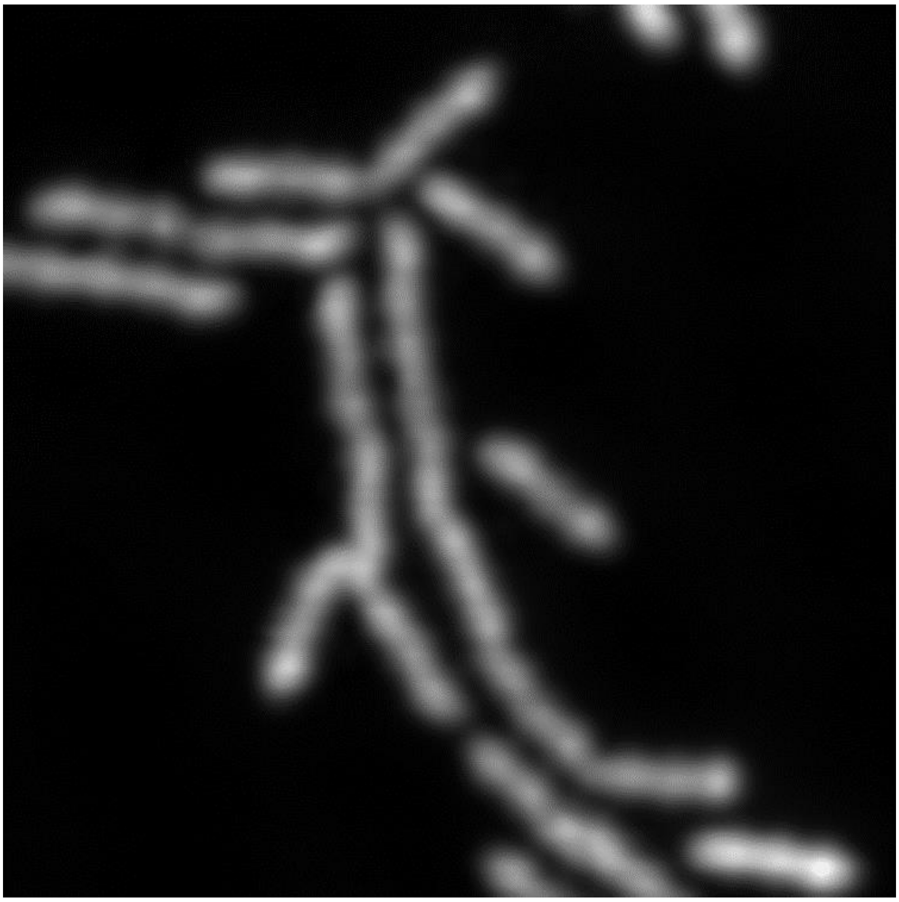

Figure S7. Constant height STM image (size: 20.8 nm x 20.8 nm) taken with a CO-terminated tip at V = 1000 mV. Single precursor molecules and chains of different length can be seen.

Slow deposition on a hot surface

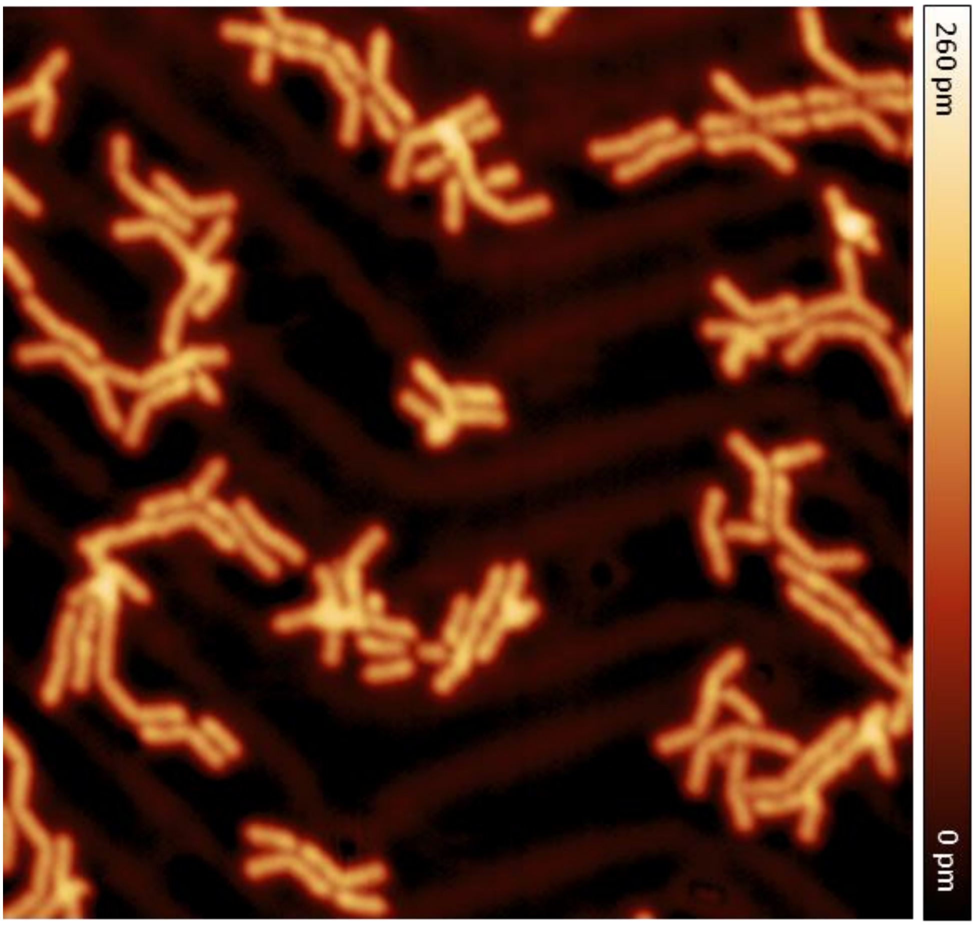

Figure S8. Overview STM image (size: 30.4 nm x 30.4 nm) after slow deposition on a 240°C hot surface. The STM image is taken under V = 500 mV and I = 5 pA.

Another approach was attempted by depositing the precursors slowly while the annealing temperature is maintained at 240°C because of the high desorption rate observed in the stepwise annealing preparation[7,8]. Overview image (Figure S8) and the corresponding statistical overview (Figure S8) of oligomer length show that a slightly larger amount of longer oligomers ($n \geq 3$) are generated.

Supporting statistical analysis

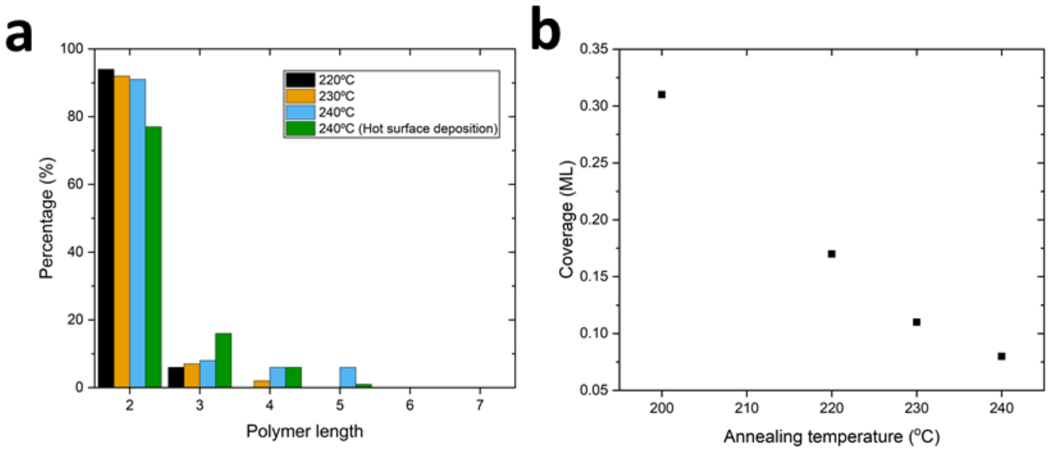

Figure S9. (a) Statistical overview of CN-vinylene-linked oligomer lengths in a simplified classification where it allows the estimation and comparison of the length distribution. This classification excludes a few kinds of structures with different shapes/conformations. A total of 604 oligomers ($n \geq 2$) are identified and categorized from different preparation temperatures. (b) Analysis of coverage (ML; area fraction) over the stepwise annealing steps.

Our results show that the dimers are the most present structures (Counts > 90%). This can be explained by the sharp coverage decrease with annealing at 200°C (Figure 1 and Figure S9b), indicating that the rate of desorption is higher than the rate of reaction (Figure S9a). Hence, part of the molecules desorb before being able to react and couple.

Close-up image of the monolayer

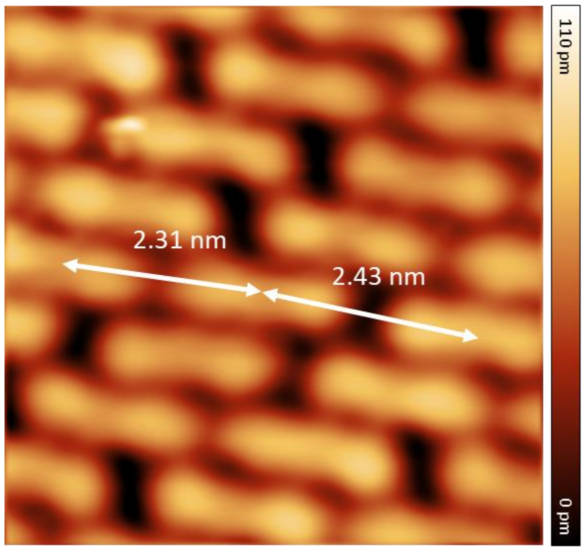

Figure S10. Close-up STM image of the monolayer islands. Different spacing between precursors is observed. STM image (size: 4.6 nm x 4.6 nm) is taken under V = 1000 mV and I = 10 pA.

Raw STM images of CN-vinylene-linked chain

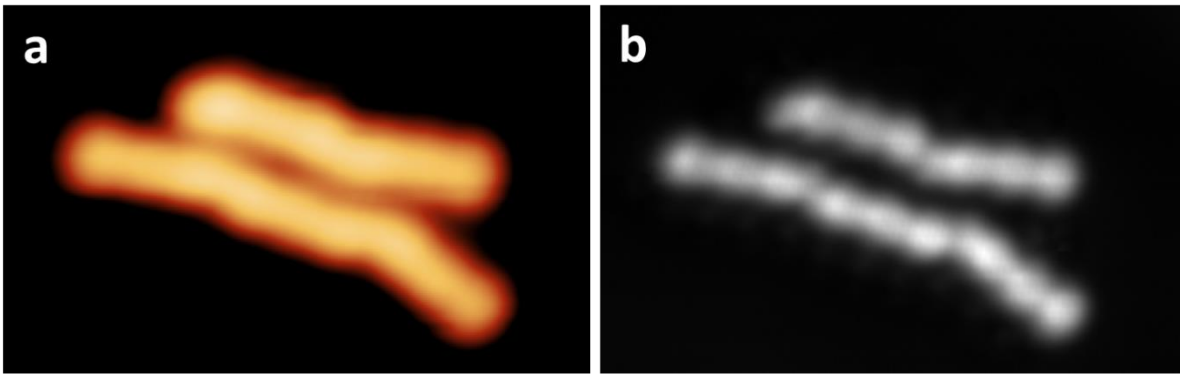

Figure S11. Raw STM images of Figure 3. (a) Constant current STM image of a phenylene vinylene trimer (size: 5.5 nm x 3.4 nm) is taken under V = 500 mV and I = 5 pA with a metallic tip and (b) the corresponding constant height high-resolution STM image (size: 5.5 nm x 3.4 nm) is taken under V = 10 mV with a CO-terminated tip.

References

1. S.-Y. Jiang, S.-X. Gan, X. Zhang, H. Li, Q.-Y. Qi, F.-Z. Cui, J. Lu and X. Zhao, *Journal of the American Chemical Society*, 2019, **141**, 14981-14986.
2. W. Fu, L. Dong, J. Shi, B. Tong, Z. Cai, J. Zhi and Y. Dong, *Macromolecules*, 2018, **51**, 3254-3263.
3. R. W. Lenz, C. E. Handlovits, *J. Org. Chem.*, 1960, **25**, 813-817.
4. N. C. Greenham, S.C. Moratti, D. D. C. Bradley, R. H. Friend, A. B. Holmes, *Natur*e, 1993, **365**, 628-630.
5. D. Becker. B. P. Biswal, P. Kaleńczuk, N. Chandrasekhar, L. Giebeler, M. Addicoat, S. Paasch, E. Brunner, K. Leo, A. Dianat, G. Cuniberti, R. Berger, X. Feng, *Chem. Eur. J.*, 2019, **25**, 6562 – 6568.
6. S. Xu, M. Richter, X. Feng, *Acc. Mat. Res.*, 2021, **2**, 4, 252-265.
7. J. Eichhorn, D. Nieckarz, O. Ochs, D. Samanta, M. Schmittel, P. J. Szabelski and M. Lackinger, *ACS Nano*, 2014, **8**, 7880-7889.
8. G. Galeotti, F. De Marchi, E. Hamzehpoor, O. MacLean, M. Rajeswara Rao, Y. Chen, L. V. Besteiro, D. Dettmann, L. Ferrari, F. Frezza, P. M. Sheverdyaeva, R. Liu, A. K. Kundu, P. Moras, M. Ebrahimi, M. C. Gallagher, F. Rosei, D. F. Perepichka and G. Contini, *Nature Materials*, 2020, **19**, 874-880.
